# Awareness and Perception About Research Ethics and Misconduct Among the Teaching Staff of Health Colleges, Jazan University, Saudi Arabia

**DOI:** 10.7759/cureus.43382

**Published:** 2023-08-12

**Authors:** Abdulrahman H Qusada

**Affiliations:** 1 Preventive Medicine, Jazan Health Affairs, Ministry of Health, Saudi Arabia, Jazan, SAU

**Keywords:** research ethics, attitude, knowledge, misconduct, saudi arabia

## Abstract

Background: Despite adopting biomedical research ethics rules, regulations, and statements, unethical behavior is pervasive and frequently reported. This study assessed the perception and awareness of research Ethics and Misconduct among the Teaching Staff of Health Colleges at Jazan University, southwest Saudi Arabia.

Purpose: This research aimed to assess the awareness and perception of research ethics and misconduct among the teaching staff of the health colleges at Jazan University in southwest Saudi Arabia.

Methods: Observational cross-sectional study targeted 424 faculty members randomly chosen from health-related colleges at Jazan University. A structured questionnaire was used to collect data using a web-based survey.

Results: The overall level of knowledge about medical ethics indicated that more than half of respondents (57.8%) had a good level of knowledge, 23.4% had a fair level of knowledge, and only 18.4% had poor knowledge. The Saudi National Committee on Bioethics was only known by 49.5% of the study participants. In addition, just 48.3% of respondents knew Saudi Arabia's national standards of ethics and principles for conducting scientific research. The knowledge of research ethics was found to be significantly (P<0.05) higher in participants with higher academic positions, such as professors, MD degree holders, and participants with more extensive work experience. About 48.2% reported that the severity of penalties for scientific misconduct would highly affect scientific integrity, and 74.3% found that their understanding of rules and procedures related to scientific misconduct has a very high effect on scientific integrity. Poor knowledge of research ethics is typically associated with an individual's academic job title, as "lecturer" showed a lower level of knowledge in comparison to other job titles. Even though about 50% of participants in the study had a good understanding of research ethics, it's crucial to ensure that all faculty members have a universal understanding. Therefore, it's essential to implement an appropriate research ethics training program.

## Introduction

Ethics in research is described as moral principles and appropriate conduct to safeguard human rights while conducting research. The entity responsible for ensuring that human subject analysis is carried out by the ethics code's ethical and moral criteria is represented by the Research Ethics Committee (REC) [1]. Additionally, RECs are required by international norms to examine research [2,3]. When researchers study, they adhere to a few ethical guidelines [4].

These guidelines are designed to safeguard participants' privacy, protect them from bodily and psychological injury, and shield them from distress and suffering. Participants must also be made aware of their needs while maintaining their anonymity and confidentiality. Participants' sensitive and private information must be protected by researchers. Deception is not permitted, though, because it is improper and could upset the participants. They must have the freedom to leave anytime it is inconvenient for them [5].

While medical research has grown significantly in underdeveloped nations during the past 10 years, there are concerns that neither the ethical framework nor the laws have kept up with this growth. Practical ethics review mechanisms and national regulations may be more ideal and, in some cases, absent in the poor world, particularly the Middle East, both at the individual and institutional levels. Numerous investigations have revealed that roughly 25% of Middle Eastern researchers filed funding requests without ethical approval [6,7].

Given the rise in human subject research both internationally and in Saudi Arabia, the study's main focus was on research ethics. But there is little research on research ethics in Jazan, and there aren't many studies on this subject in Saudi Arabia. The limited study raises concerns regarding the comprehension and application of research ethics concepts as well as awareness of national and international ethical guidelines [8]. Therefore, the purpose of this study was to evaluate how the teaching staff of the health colleges at Jazan University in southwest Saudi Arabia perceived and were aware of research ethics and misconduct.

## Materials and methods

Study design and setting and population

A cross-sectional study was conducted at Jazan University at the colleges of medicine, dentistry, pharmacy, and applied medical sciences between March 1, 2023 and May 31, 2023. The university is in the Jazan region in the south of Saudi Arabia. All teaching staff of medicine, dentistry, pharmacy, and applied medical sciences colleges at Jazan University were eligible to participate in this study.

Sampling procedures

The participant was chosen using a random sampling method. According to the following equation ($n=Z^2 P(1- P)/ D2$), which assumes that the percentage of people with strong knowledge is 50% with a 95% confidence interval (CI) and a 0.05 margin of error, the minimal sample size was 384. The authors added 10% to the sample as compensation for the possible non-response, so 424 participants were enrolled. The teaching staff was selected by simple random technique from the colleges of medicine and dentistry (116 from each faculty), the College of Pharmacy (80), and the College of Applied Medical Sciences (112). The inclusion criteria were any teaching staff who worked in Jazan University at the previously mentioned colleges who agreed to participate.

Inclusion Criteria

All teaching staff who worked at Jazan University's colleges of medicine, dentistry, pharmacy, and applied medical sciences were eligible to participate if they agreed to do so.

Exclusion Criteria

Teaching staff who did not work at the above-mentioned colleges or who declined to participate were excluded from the study.

Data collection tools

An anonymous, self-administered, validated questionnaire was used to gather the data. It was based on data that was taken from an earlier study [9]. Part one of the questionnaire, which consists of eight questions, asks participants about their personal and socio-demographic backgrounds as well as their familiarity with the rules governing research ethics. Additionally, part two (25 questions) evaluates the understanding of the fundamentals of research ethics. Part three's 10 questions also take the attitude toward research ethics into account. The perception of scientific misconduct in the workplace is measured in part four (18 questions), which is the last portion.

To ensure content validity, the questionnaire was reviewed by specialists and committee members, as well as through pretesting and cognitive debriefing. Cronbach's coefficient alpha was used to estimate internal consistency reliability coefficients for the initial and the retest tool administrations in order to ensure the reliability of the questionnaire. A pilot study involving 30 participants who were not included in the survey was conducted to evaluate the face validity and the internal consistency reliability. It was discovered that the tool's median Cronbach's alphas (for the original and retest) were higher than 0.82. Additionally, test-retest reliability over a two-week period was evaluated, and Pearson product-moment correlations between the first and retest administrations were obtained. Correlation coefficients (r) > 0.70 were observed (P 0.01).

Participants answered 25 questions about the rules and norms of research ethics to gauge their knowledge of the subject. Three points were awarded for each successfully answered question, zero for each incorrect response, and one point for each correct response. The overall score for the knowledge questions ranged from 0 to 75; the scores for weak knowledge were from 0 to 25, fair knowledge from 26 to 50, and strong knowledge from 51 to 75. Participants were also asked ten questions about their attitudes, with possible responses ranging from highly agree to agree, uncertain, disagree, and strongly disagree. While the negative attitude decreased by one, the positive attitude increased by five. Positive attitudes scored between 38 and 50, whereas negative attitudes scored between 10 and 23, and in-between ranged between 24 and 37.

Data analysis

Data analysis was conducted using the Statistical Package for Social Sciences (SPSS) version 23 for Windows. The descriptive statistics were calculated as frequency, counts, percentages, mean, and SD. The chi-square was used to test the statistical significance of associations between study variables. Logistic regression analysis was performed to identify factors associated with poor knowledge and negative attitude. P-value < 0.05 was considered statistically significant.

Statement of the Institutional Review Board

The Jazan University's standing committee for scientific research provided official ethical approval under the reference number REC-44/07/551 (HAPO-10-Z-001). Data collection was accepted to be contingent upon consent. All information gathered was kept private and solely used for the study.

## Results

A total of 424 teaching staff took part in the present work, with a response rate of 100.0%. Male participants represented 63.7%. The mean age of the participants ± SD was 44 ± 10.01 years. Regarding the qualifications, 7.3% of participants had a master’s degree, while 44.6% and 48.1% had PhD and MD degrees, respectively. Assistant professors were 66.5%, while only 5.7% were professors. About 27.4% of the participants belong to either faculty of medicine or dentistry, 18.8% to the faculty of pharmacy, and 26.4% to the applied medical science college. Moreover, 19.3% have work experience of less than five years, and 32.6% have work experience of more than ten years. Nearly 84.7% of the participants have research projects that included human subjects, and 92.5% have research projects which included human biological samples (e.g., blood, tissue, urine, teeth, saliva, etc.) (Table 1).

**Table 1:** Personal and socio-demographic background of the studied sample

Variables		n	%
Gender	Male	270	63.7
	Female	154	36.3
Age in year	Mean ± S.D = 44 ± 10.01		
Qualification	Master	31	7.3
	PhD	189	44.6
	MD	204	48.1
Academic job title	Professor	24	5.7
	Associate Professor	87	20.5
	Assistant Professor	282	66.5
	Senior Lecturer/Lecturer	31	7.3
The College	Medicine	116	27.4
	Dentistry	116	27.4
	Pharmacy	80	18.8
	Applied medical sciences	112	26.4
Years of experience as a teaching staff	<5	82	19.3
	5-10	204	48.1
	>10	138	32.6
Did your research projects include human subjects?	Yes	359	84.7
	No	65	15.3
Did your research projects include human biological samples	Yes	392	92.5
	No	32	7.5

**Figure 1:**
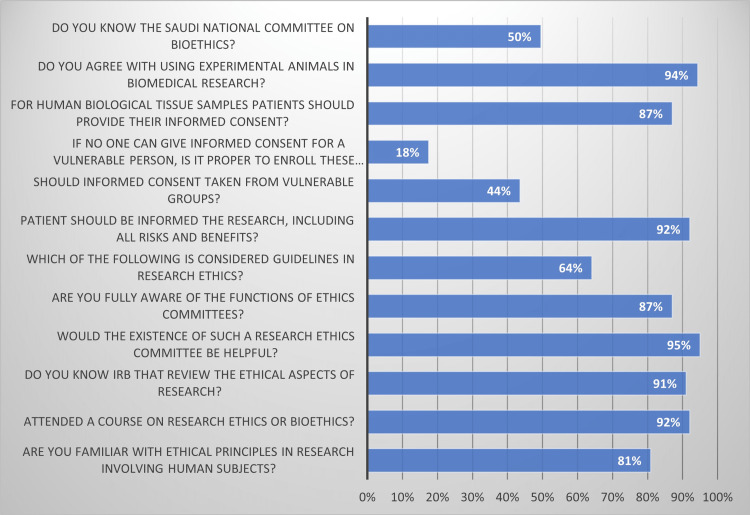
Knowledge of the faculty members regarding research ethics (participants with yes response)

The participants' familiarity with research ethics is the study's primary outcome variable. Approximately, 80% of participants are found to be familiar with the ethical rules that govern using human subjects in research; 4.7% are members of a REC; 92.0% have taken a research ethics or bioethics course; 60.4% report that the university offers a research ethics training program; and 91.0% believe that having a REC would be beneficial. Additionally, 69.1% of participants recognize the function of the REC, and 62.5% of participants are aware of the significance of ethics and the rules governing research ethics. It also reveals that 92.0% of participants believe that patients should be told about all the specifics of the research, including any risks and advantages, and that 95.5% believe that patients should always be required to sign a written consent form in order to give their informed consent. The table also reveals that 87.0% of respondents believe that patients should be educated about using human biological tissue samples, including solid tissues, blood, and other bodily fluids. Additionally, roughly 94.3% of participants support utilizing animals in experiments for biomedical research, and 100.0% of participants are aware that laws in Saudi Arabia govern research ethics. While only 48.3% are aware of the Saudi Arabian national codes of ethics and guiding principles for scientific research, 49.5% are aware of the Saudi National Committee on Bioethics (Figure 1).

The overall level of knowledge about medical ethics indicated that more than half of respondents (57.8%) had a good level of knowledge, 23.4% had a fair level of knowledge, and only 18.4% had poor knowledge. The degree of knowledge regarding ethics increases with the increase in age of the participants to become maximally (80.5%) in older than 50 years and with experience of more than 10 years (68.8%). A reasonable degree of knowledge is more in males (67.0%), associate professors (85.1%), and members who got MD (71.1%). The distribution of different degrees of knowledge according to the age groups, gender, qualification, job title, and years of experience is of statistically significant difference (P < 0.05) (Table 2).

**Table 2:** Distribution of the respondents’ degree of knowledge according to their general characteristics CI: Confidence interval

Variable		Poor		Fair		Good		χ^2 ^_(P-value)_
		No	%	No	%	No	%	
Age groups [in years]								
	< 40	52	44.8	36	31.0	28	24.2	112.7 (P < 0.001)
	40- 50	14	9.4	46	30.9	89	59.7	
	> 50	12	7.6	19	11. 9	128	80.5	
Gender								
	Male	41	15.2	48	17.8	181	67.0	26.6 (P < 0.001)
	Female	37	24.0	53	34.4	64	41.6	
Qualification								
	Master	14	42.2	12	38.7	5	16.1	44.1 (P < 0.001)
	PhD	42	22.2	52	27.5	95	50.3	
	MD	22	10.8	37	18.1	145	71.1	
Academic job title								
	Senior lecturer/lecturer	18	58.1	7	22.6	6	19.3	67.3 (P < 0.001)
	Assistant professor	48	17.0	84	29.8	150	53.2	
	Associate professor	8	9.2	5	5.7	74	85.1	
	Professor	4	16.7	5	20.8	15	62.5	
The college that participants belong to								
	Medicine	18	15.5	27	23.3	71	61.2	13.0 (P < 0.05)
	Dentistry	15	12.9	35	30.2	66	56.9	
	Pharmacy	23	28.8	19	23.7	38	47.5	
	Applied Medical Sciences	22	19.6	20	17.9	70	62.5	
Years of experience as a teaching staff								
	< 5	39	47.6	25	30.5	18	21.9	72.1 (P < 0.001)
	5- 10	24	11.8	48	23.5	132	64.7	
	> 10	15	10.9	28	20.3	95	68.8	
The overall level of knowledge								
		78	18.4	101	23.4	245	57.8	
95% CI for the level of knowledge								
		15.0-22.4		20.0-28.1		53.0-62.4		

The most important predictors of poor knowledge of research ethics include academic job title (senior lecturer/lecturer) (odds ratio (OR) = 5.56) and years of experience as a teaching staff < 5 years (OR= 5.18). Moreover, master qualification (OR = 4.62) and age < 40 years (OR = 4.14) are predictors of poor knowledge. Finally, the college of pharmacy (OR = 3.21) predicts poor knowledge (Table3).

**Table 3:** Multivariate regression analysis for predictors of poor knowledge of Research Ethics

Predictors	OR	95% CI	p-Value
Age (< 40 y) (reference: > 50 years)	4.14	1.32- 1.87	<0.0001
Gender (females) (reference: males)	2.18	0.55- 1.42	> 0.05
Qualification (Master) (reference: MD)	4.62	1.12- 1.65	<0.0001
Academic job title (senior lecturer/lecturer) (reference: Professor)	5.56	1.32- 1.82	<0.0001
The college that participants belong to (Pharmacy) (reference: Medicine)	3.21	1.12- 1.65	<0.0001
Years of experience as a teaching staff: (< 5) (reference: > 10 Y)	5.18	1.37- 1.89	<0.0001

The distribution of participants’ attitudes towards research ethics was measured according to the socio-demographic characteristics in Table 4. The overall level of attitude showed that about 74.7% of the participants have a positive attitude toward research ethics, while only 5.7% have a negative attitude. According to the table, there is a significant difference (p < 0.05) in the level of attitude according to age, where 87.9% and 86.2% of participants who belonged to the age group (40-50) and those who were older than 50 years, respectively, had positive attitudes, while 46.6% of those who were younger than 40 years had in-between. Regarding gender, it was reported that 69.7% and 83.8% of males and females had positive attitudes, and the distribution of different degrees of attitude according to gender was statistically significant (p < 0.05). Moreover, about 73.0% and 81.4% of those who had PhD and MD, respectively, had positive attitudes, and the distribution of different degrees of attitude according to qualification’s degree was statistically significant (p < 0.05). The table further indicated that there a significant difference in the level attitudes regarding academic job, college and years of experience s (p value <0.05 for all) (Table 4).

**Table 4:** Distribution of the respondents’ degree of attitude according to their general characteristics CI: Confidence interval

Variable		Degree of attitude						Χ^2 ^_(p-value)_
		Negative		In-between		Positive		
		No	%	No	%	No	%	
Age groups [in years]								
	< 40	13	11.2	54	46.6	49	42.2	91.05 (P < 0.001)
	40- 50	6	4.0	12	8.1	131	87.9	
	> 50	5	3.1	17	10.7	137	86.2	
Gender								
	Male	16	5.9	66	24.4	188	69.7	11.72 (P < 0.001)
	Female	8	5.2	17	11.0	129	83.8	
Qualification								
	Master	5	16.2	13	41.9	13	41.9	25.53 (P < 0.001)
	PhD	8	4.2	43	22.8	138	73.0	
	MD	11	5.4	27	13.2	166	81.4	
Academic job title								
	Senior lecturer/lecturer	6	19.4	8	25.8	17	54.8	44.40 (P < 0.001)
	Assistant professor	9	3.2	38	13.5	235	83.3	
	Associate professor	5	5.7	28	32.2	54	62.1	
	Professor	4	16.7	9	37.5	11	45.8	
College								
	Medicine	5	4.3	30	25.9	81	69.8	0.11 (P < 0.05)
	Dentistry	7	6.0	27	23.3	82	70.7	
	Pharmacy	7	8.8	11	13.8	62	77.4	
	Applied Medical Sciences	5	4.5	15	13.4	92	82.1	
Years of experience								
	< 5	8	9.8	38	46.3	36	43.9	53.22 (P < 0.001)
	5- 10	10	4.9	27	13.2	167	81.9	
	> 10	6	4.4	18	13.0	114	82.6	
The overall level of attitude								
		24	5.7	83	19.6	317	74.7	
95% CI for the level of attitude								
		03.8-08.3		16.1-23.6		70.4-78.7		

Table 5 displays how participants felt about scientific misbehavior in terms of research ethics. The table shows that approximately 76.4% of the participants occasionally see plagiarism, 82.3% rarely see data fabrication, 49.5% never see intentional protocol violations related to subject enrollment, 48.2% never see the selective erasure of data from 'outlier' cases, and 71.2% never see disagreements over authorship. Additionally, nearly 91.3% believed that the principal investigator alone is responsible for a study's scientific integrity, 97.2% believed that all professional education programs should include information about standards of research ethics, 66.3% disagreed that they felt uncomfortable discussing unethical behavior with researchers, and 85.8% disagreed that dishonesty and data misrepresentation are accepted in society and do no harm. (Table 5).

**Table 5:** Perception of the participants towards scientific misconduct in research ethics

	How much you witness the following:	Never	Seldom	Occasionally	Frequently
		No (%)	No (%)	No (%)	No (%)
1	Plagiarism	7 (1.7)	15 (3.5)	324 (76.4)	78 (18.4)
2	Falsifying data (changing or omission of research results)	0 (0.0)	192 (45.3)	130 (30.7)	102 (24.0)
3	Fabricating data (making up data)	9 (2.1)	349 (82.3)	37 (8.8)	29 (6.8)
4	Intentional protocol violations related to subject enrollment	210 (49.5)	137 (32.3)	64 (15.1)	13 (3.1)
5	The selective dropping of data from ‘outlier’ cases	204 (48.2)	188 (44.3)	32 (7.5)	0 (0.0)
6	Falsification of bio sketch, resume, reference list	139 (32.8)	206 (48.6)	63 (14.9)	16 (3.7)
7	Disagreements about authorship	302 (71.2)	103 (24.3)	19 (4.5)	0 (0.0)
8	Pressure from the study sponsor (e.g., a pharmaceutical company or device company) to engage in unethical practices	424 (100.0)	0 (0.0)	0 (0.0)	0 (0.0)
	What do you think about the following situations?	Agree	Disagree	I don’t know	
		No (%)	No (%)	No (%)	
9	I think the responsibility for the scientific integrity of a study lies with the principal investigator only	387 (91.3)	28 (6.6)	9 (2.1)	
10	All professional education programs should include information about standards of research ethics	412 (97.2)	0 (0.0)	12 (2.8)	
11	I feel uncomfortable talking with researchers about unethical behavior	137 (32.3)	281 (66.3)	6 (1.4)	
12	Dishonesty and misrepresentation of data are expected in society and do not hurt anybody	35 (8.3)	364 (85.8)	25 (5.9)	

**Figure 2:**
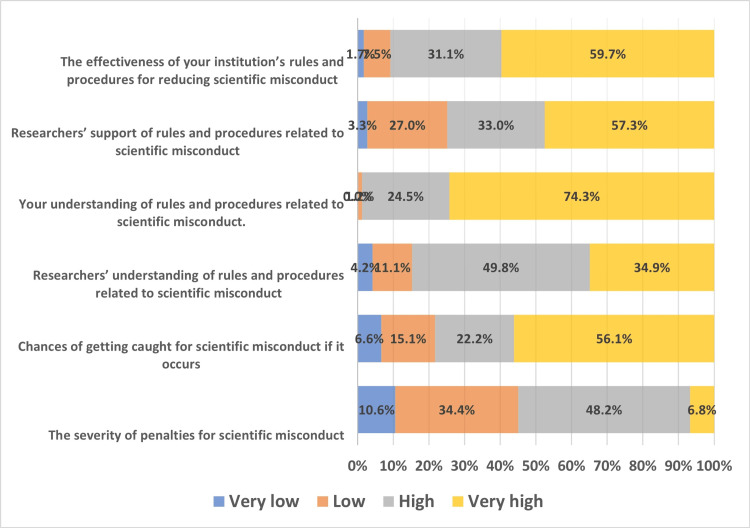
Perception of the participants towards scientific misconduct in research ethics.

In terms of how the participants perceived scientific misconduct in research ethics, it was discovered that about 48.2% of the participants believed that the severity of the penalties for such misconduct would have a significant impact on scientific integrity and that 74.3% believed that their understanding of the policies and procedures relating to such misconduct has a significant impact as well. 59.7% discovered that scientific integrity is highly impacted by how well one's institution's policies and procedures for preventing scientific misconduct work (Figure 2).

## Discussion

In the last 50 years, ethics have become increasingly significant in medicine. Researchers were once seen to have high morals and to be more trustworthy and fairer than the general population. To keep the patient's trust and the standard of treatment high, stronger and more in-depth knowledge of research ethics principles, such as respect for autonomy, safety, and the social benefit of the research conclusion, is now required [3]. The reporting of misconduct is rising, but it can be challenging to determine whether these data are accurate. One out of every 100,000 scientists said and reported engaging in fraud, according to data acquired from the US government, and one out of every 10,000 scientists, according to a different report [10,11]. On the other side, PubMed reports that article retractions occur 0.002% of the time. The literature has studies with frequency speculations ranging from 0.02 to 0.2%, but these are false [12]. 2% of clinical researchers were found guilty of serious scientific misconduct between 1977 and 1990, according to statistics drawn from reports submitted to the US Food and Drug Administration (FDA) [13].

In the Middle East, particularly in Saudi Arabia, human subject research is steadily growing. Even though great efforts have been made, only a few nations have produced their national research standards, according to Alahmad, Al-Jumah, and Dierickxthe [14]. In order to conduct research on living things in the Saudi Arabian context without breaking any laws, the Law of Ethics of Research on Living Things (Article 321, by the Ministers' Council to the King of Saudi Arabia in 2010) and the implementing regulations of the Law of Ethics of Research on Living Things, which was issued on December 25, 2011, aim to establish a foundation and necessary rules [15].

As a result, there is a critical need for data collection to evaluate the clinical researchers' knowledge, the reliability of their study, and their ethical behavior in order to develop high-quality, reliable, evidence-based research. Despite several cases, Fanelli's systematic review and meta-analysis study on research ethics and misconduct ignored any Middle Eastern analysis, despite the pressing need for such a study to be conducted in Saudi Arabia [16].

The current study's findings are focused on research ethics training in Saudi Arabia in relation to both worldwide standards and local codes of ethics.

Only one study examined Egyptian faculty members' attitudes and knowledge of research ethics in the Middle East [5]. Contrary to our findings, the authors' research indicated that the average physician's knowledge score was 42%, whereas our study employed a non-parametric approach. The results of the current study showed that the majority of participants (57.8%) had good knowledge, while only a small minority (18.4%) had poor knowledge. The awareness of research ethical rules is correlated with qualification, professional rank (job title), years of experience, and prior research ethics training, much as a study done in Lebanon on physicians [9]. Contrary to our findings, the experience in the Egyptian study was not linked to awareness. In previous studies, participants expressed concern about the potential for research misconduct at a rate of 77.3%, and 71.8% of them were aware of the laws governing research ethics [17,18]. Even though few doctors receive adequate training in research ethics, it is reasonable to expect that qualified doctors will be familiar with the guidelines in addition to their clinical competence [19]. Many participants (74.7%) in the current survey had good attitudes toward research ethics; this finding is consistent with that of other studies [5,9]. The fact that academics and staff had participated in seminars or workshops on research ethical guidelines may help to explain this.

Participants in this study were questioned about any instances of scientific misconduct they may have seen at work. Notably, a sizable proportion of participants reported seeing instances of scientific misconduct in the following order: Many of them (76.4%) occasionally saw plagiarism, only 45.3% rarely saw data falsification, most of them (82.3%) rarely saw data fabrication, only 49.5% and 48.2% never saw the selective dropping of data from outlier cases, and most of them (71.2%) never saw disagreement over authorship. In a related Lebanese research, numerous doctors said that they had seen instances of scientific misconduct, including plagiarism, disputed authorship, and the fabrication and falsification of data [9]. In a comparable Norwegian poll, 27% of doctors said they were aware of unethical behavior in their universities' research, but 42% said that the information was not made public [20]. In addition, a higher rate of misbehavior was noted in a global survey of biostatisticians (51%), as well as in British medical consultants (56%) [21, 22]. Nearly 14.12% of doctors acknowledged seeing falsification in a meta-analysis of surveys involving self-reported and observed scientific misconduct, and up to 72% saw other dubious research methods [16]. Another study revealed that many doctors frequently observed one or more instances of scientific misconduct at work [23].

Additionally, participants were asked how various factors could impact scientific integrity; the responses varied in frequency, with 48.2% of participants believing that the severity of penalties for scientific misconduct has a high effect, the majority (56.1%) believing that chances of being discovered for such misconduct, should it occur, and 49.8% believing that researchers' understanding of rules and procedures related to such misconduct has a very high impact. These results are consistent with those from other studies [9].

The current study also showed that the academic job title (senior lecturer/lecturer), years of experience as a teaching staff less than five years, master qualification, age less than 40 years, and the college that participants belong to (Pharmacy) are the most significant predictors of poor knowledge of research ethics. The knowledge was higher among professors who had more years of experience and, of course, were older in age. Regarding the college that the participants belong to, the knowledge was higher among participants who used human subjects in their biomedical research. These findings are unique because no other study has addressed this issue. This explains why participants from pharmacy colleges had less research knowledge.

Communities are currently losing trust due to misconduct in research and the lack of responsibility among biomedical researchers [24]. They emphasize upholding the dignity and autonomy of human subjects, as well as the integrity of the study findings. As a result, there is a link between ethical standards and public confidence in biomedical research. The community's confidence in biomedical research and its critical role in furthering scientific understanding would be adversely affected by any research misconduct. By improving researchers' understanding of the ethics principles, this public issue may be stimulated and addressed.

Finally, we note that there may be some limitations to this study. The questions in this cross-sectional study, which is based on a small sample, may only touch on a few rules and principles of research ethics, but they nonetheless represent the fundamental knowledge that teaching staff members should have before engaging in any biomedical research involving human subjects.

## Conclusions

In conclusion, our study showed that Jazan University's teaching staff in health colleges has a good understanding of the rules and procedures governing research ethics, and they also have favorable opinions about these practices. In addition, professional position (job title), education, and experience length all have a beneficial influence on participants' development of research ethics knowledge. Most participants reported being aware of research ethical guidelines, which indicates greater knowledge. Additionally, a strong correlation between participants' age, gender, and college affiliation and their awareness of research ethical regulations was discovered. Results revealed that our participants perceived high rates of research misconduct and scientific misbehavior, with over half of the physicians reporting having seen scientific misconduct at some point in their employment. In line with and concurrent with international efforts to improve researchers' knowledge of research ethics principles, our study also highlighted the significance of bolstering the function and role of RECs in developing and establishing institutional and national educational programs in research ethics.

## References

[1] DePoy E, Gitlin LN (2016). Introduction to research understanding and applying multiple strategies.

[2] (2023). Standards and operational guidance for ethics review of health-related research with human participants. https://www.who.int/publications/i/item/9789241502948.

[3] Rana J, Dilshad S, Ahsan MA (2021). Ethical issues in research. Global Encyclopedia of Public Administration, Public Policy, and Governance.

[4] Heale R, Shorten A (2017). Ethical context of nursing research. Evid Based Nurs.

[5] El-Dessouky HF, Abdel-Aziz AM, Ibrahim C, Moni M, Abul Fadl R, Silverman H (2011). Knowledge, awareness, and attitudes about research ethics among dental faculty in the Middle East: a pilot study. Int J Dent.

[6] Mallela KK, Walia R, TM CD, Das M, Sepolia S, Sethi P (2015). Knowledge, attitudes and practice about research ethics among dental faculty in the North India. J Int Oral Health.

[7] Cox DJ, Suarez VD, Marya V (2023). Ethical principles and values guiding modern scientific research. Research Ethics in Behavior Analysis.

[8] Ashurst C, Barocas S, Campbell R, Raji D (2022). Disentangling the components of ethical research in machine learning. FAccT '22: Proceedings of the 2022 ACM Conference on Fairness, Accountability, and Transparency.

[9] Azakir B, Mobarak H, Al Najjar S, El Naga AA, Mashaal N (2020). Knowledge and attitudes of physicians toward research ethics and scientific misconduct in Lebanon. BMC Med Ethics.

[10] Steneck NH (2006). Fostering integrity in research: definitions, current knowledge, and future directions. Sci Eng Ethics.

[11] Krimsky S (2007). When conflict-of-interest is a factor in scientific misconduct. Med L.

[12] Claxton LD (2005). Scientific authorship. Part 1. A window into scientific fraud?. Mutat Res.

[13] Glick JL (1992). Scientific data audit—a key management tool. Account Res.

[14] Alahmad G, Al-Jumah M, Dierickx K (2012). Review of national research ethics regulations and guidelines in Middle Eastern Arab countries. BMC Med Ethics.

[15] Majid IA, AlKashgari RH, AlYahya AA, Alikutty FK, Rahman SM (2019). Developing and establishing research guidelines in a private higher education institution of Saudi Arabia. An experience. Saudi Med J.

[16] Fanelli D (2009). How many scientists fabricate and falsify research? A systematic review and meta-analysis of survey data. PLoS One.

[17] Ogunrin O, Ogunrin OA, Murray BJ (2016). Knowledge and practice of research ethics among biomedical researchers in southern Nigerian tertiary institutions. J Clin Res Bioeth.

[18] Kandeel N, El-Nemer A, Ali NM (2011). A multicenter study of the awareness and attitudes of Egyptian faculty towards research ethics: a pilot study. J Empir Res Hum Res Ethics.

[19] Moazam F (2000). Families, patients, and physicians in medical decisionmaking: a Pakistani perspective. Hastings Cent Rep.

[20] Hals A, Jacobsen G (1993). Dishonesty in medical research. A questionnaire study among project administrators in Health Region 4 (Article in Norwegian). Tidsskr Nor Laegeforen.

[21] Ranstam J, Buyse M, George SL (2000). Fraud in medical research: an international survey of biostatisticians. Control Clin Trials.

[22] Geggie D (2001). A survey of newly appointed consultants’ attitudes towards research fraud. J Med Ethics.

[23] Okonta PI, Rossouw T (2014). Misconduct in research: a descriptive survey of attitudes, perceptions and associated factors in a developing country. BMC Med Ethics.

[24] Harkness J, Lederer SE, Wikler D (2001). Laying ethical foundations for clinical research. Bull World Health Organ.

